# SGCE promotes breast cancer stemness by promoting the transcription of FGF-BP1 by Sp1

**DOI:** 10.1016/j.jbc.2023.105351

**Published:** 2023-10-12

**Authors:** Ting Qiu, Lei Hou, Lina Zhao, Xinye Wang, Zhongmei Zhou, Chuanyu Yang, Huifeng Zhang, Dewei Jiang, Baowei Jiao, Ceshi Chen

**Affiliations:** 1 Key Laboratory of Animal Models and Human Disease Mechanisms of Chinese Academy of Sciences, KIZ-CUHK Joint Laboratory of Bioresources and Molecular Research in Common Diseases, Kunming Institute of Zoology, Kunming, Yunnan, China; 2 Kunming College of Life sciences, University of Chinese Academy Sciences, Kunming, Yunnan, China; 3 Department of Breast Disease, Henan Breast Cancer Center, Affiliated Cancer Hospital of Zhengzhou University & Henan Cancer Hospital, Zhengzhou, China; 4 State Key Laboratory of Genetic Resources and Evolution, Kunming Institute of Zoology, Chinese Academy of Sciences, Kunming, Yunnan, China; 5 Department of Clinical Pharmacy, The First People’s Hospital of Yunnan Province/The Affiliated Hospital of Kunming University of Science and Technology, Kunming, China; 6 Academy of Biomedical Engineering, Kunming Medical University, Kunming, China; 7 The Third Affiliated Hospital, Kunming Medical University, Kunming, China

**Keywords:** breast cancer stem cells, nuclear localization, gene transcription, TNBC

## Abstract

Breast cancer stem cells are mainly responsible for poor prognosis, especially in triple-negative breast cancer (TNBC). In a previous study, we demonstrated that ε-Sarcoglycan (SGCE), a type Ⅰ single-transmembrane protein, is a potential oncogene that promotes TNBC stemness by stabilizing EGFR. Here, we further found that SGCE depletion reduces breast cancer stem cells, partially through inhibiting the transcription of FGF-BP1, a secreted oncoprotein. Mechanistically, we demonstrate that SGCE could interact with the specific protein 1 transcription factor and translocate into the nucleus, which leads to an increase in the transcription of FGF-BP1, and the secreted FBF-BP1 activates FGF-FGFR signaling to promote cancer cell stemness. The novel SGCE-Sp1-FGF-BP1 axis provides novel potential candidate diagnostic markers and therapeutic targets for TNBC.

Breast cancer has become the leading cause of cancer incidence among women aged 20 to 59 years worldwide, and the incidence and mortality rate of breast cancer were the highest in most countries among women in 2020 (1). Basal-like breast cancer is an aggressive molecular subtype that represents 10 to 25% of all breast cancers, and it make up approximately 50 to 75% of the triple-negative breast cancer (TNBC) (2). This subtype lacks the expression of estrogen receptor α, progesterone receptor, and human epidermal growth factor 2 (2, 3, 4). However, due to the lack of effective therapeutic targets (such as estrogen receptor α and human epidermal growth factor 2), conventional chemotherapy is still the primary established treatment for TNBC patients (3, 4). It is urgent to develop more targeted therapies in TNBC.

Breast cancer stem cells (BCSCs) are a small subpopulation of self-renewing cancer cells responsible for drug resistance, cancer initiation, and cancer progression (5, 6). Generally, identification of BCSCs from tumor samples or breast cancer cell lines has been based mainly on CD44^+^/CD24^−/low^ or high aldehyde dehydrogenase 1 activity (5, 6). Although chemotherapy can kill breast cancer cells, it fails to eliminate the BCSCs population of TNBC, which may be the reason for recurrence and drug resistance of TNBC patients (7). Thus, targeting BCSCs is the key to improving the efficacy of TNBC because they initiate tumor growth.

ε-Sarcoglycan (SGCE) is a member of the sarcoglycan family and includes transmembrane components in a dystrophin–glycoprotein complex (8). It was hypothesized that defects in the dystrophin–glycoprotein complex disrupt the mechanical link between the cytoskeleton of muscle and the extracellular matrix (9). SGCE is widely expressed in several different tissue types compared with other sarcoglycan family members (9). Mutations in the gene encoding SGCE cause myoclonus–dystonia syndrome (10). Although SGCE was reported to be a cancer risk gene in gastric cancer (11, 12), colorectal cancer (13), hepatocellular carcinoma (14, 15), and B-cell chronic lymphocytic leukemia (16), how it participates in cancer remains elusive. SGCE was found to be highly expressed in undifferentiated human embryonic stem cells lines and downregulated in differentiated derivatives (17), suggesting that SGCE may regulate stemness. In our previous study, we analyzed the expression profile in BCSC populations of TNBC at single-cell resolution based on published single-cell RNA-Seq data and found 19 highly and commonly expressed genes including SGCE (18). Indeed, our previous study showed that the expression of SGCE was positively correlated with the highly expressed genes identified in the CD24^low^CD44^high^ or aldehyde dehydrogenase 1^+^ populations (18). Furthermore, we showed that SGCE promotes cancer stemness though the EGFR-AKT axis in TNBC (18). However, EGFR could only partially restore the function of SGCE knockdown; therefore, the molecular mechanisms of SGCE involved in the TNBC stemness are insufficiently understood.

Fibroblast growth factor binding protein 1 (FGF-BP1) is a secreted, heparin-binding protein, that can bind fibroblast growth factor (FGF) 1 and 2 (19). These FGFs are usually stored in an inactive form on heparan sulfate proteoglycans in the extracellular matrix, and it has been proposed that FGF-BP1 functions as a chaperone molecule, that can mobilize locally stored FGF and present growth factors to its tyrosine kinase receptor (19, 20). We previously reported that FGF-BP1 is a transcription target of Krüppel-like Factor 5 (KLF5) transcription factor in basal-like breast cancer (21). The transcription factor Sp1 (specific protein 1) also belongs to the Sp/KLF transcription factor family. Sp1 binds to GC boxes with the consensus sequence 5′-G/T-GGGCGG-G/A-3′ (22, 23, 24). Sp1 promotes tumor development in several cancer types (25, 26, 27).

Here, we show that SGCE promotes breast cancer stemness partially by upregulating the expression of FGF-BP1. Mechanistically, SGCE increases Sp1 translocation into the nucleus, facilitates Sp1 binding to the FGF-BP1 gene promoter and increases FGF-BP1 mRNA expression.

## Results

### SGCE upregulates FGF-BP1 mRNA expression in TNBC cells

**Figure 1:**
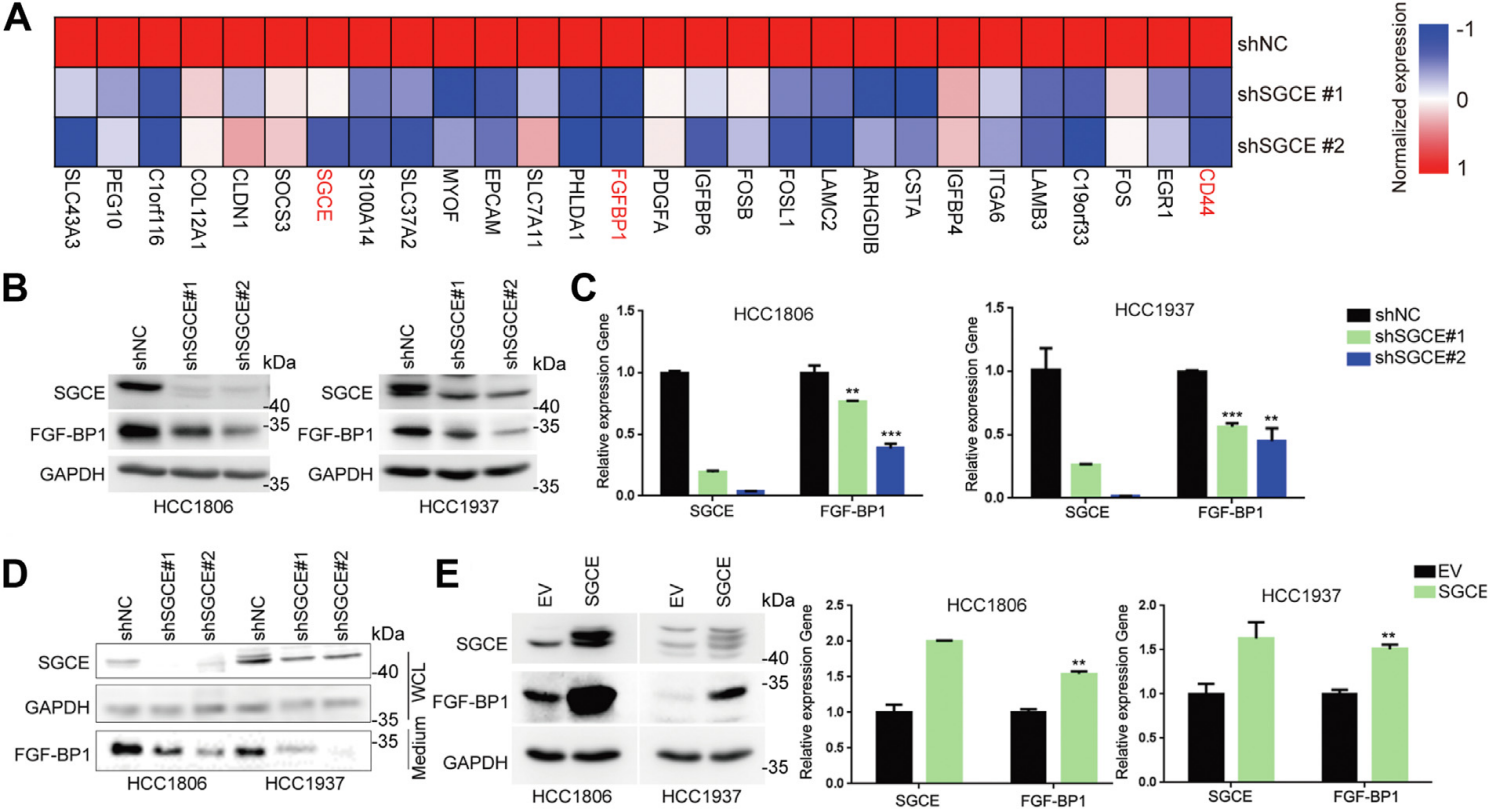
**SGCE positively regulates FGF-BP1 in TNBC cells.***A*, heatmap showing the 29 downregulated genes that were identified by RNA-seq upon SGCE silencing in HCC1806 cell. *B* and *C*, Western blot and RT-qPCR assay showed that SGCE knockdown inhibited FGF-BP1 expression in HCC1806 and HCC1937 cells. *D*, the efficiencies of SGCE knockdown in whole-cell lysates (WCL) and FGF-BP1 protein expression in cell culture medium (Medium) were examined by Western blot in HCC1806 and HCC1937 cells. *E*, Western blot and RT-qPCR assay showed that SGCE overexpression increased the FGF-BP1 mRNA and protein levels in HCC1806 and HCC1937 cells. Data are presented as the mean ± SEM. n = 3 per group. ∗∗*p* < 0.01 and ∗∗∗*p* < 0.001 *versus* shNC group. FGF-BP1, fibroblast growth factor binding protein 1; SGCE, ε-sarcoglycan; TNBC, triple-negative breast cancer.

Previously, we identified SGCE as a BCSC marker gene (18). To further confirm the involvement of SGCE in regulating TNBC stemness, SGCE-knockdown TNBC cells were used for high-throughput RNA-sequencing assays (18). RNA-sequencing data revealed that SGCE knockdown led to the downregulation of the expression of a subset of genes, including FGF-BP1 and CD44 (Fig. 1*A*). FGF-BP1 has been demonstrated to enhance the biological and biochemical activities of FGFs and to be closely related to the growth of several cancers. Thus, we suspected that SGCE might promote cancer stemness partially through the FGF-BP1 in TNBC.

We performed RT–qPCR and Western blot to confirm the RNA-sequencing results in HCC1806 and HCC1937 cells. Indeed, knockdown of SGCE dramatically decreased the expression of FGF-BP1 at the mRNA and protein levels (Fig. 1, *B* and *C*). We also detected the secreted FGF-BP1 in cell condition media and found that FGF-BP1 secretion was decreased upon SGCE knockdown (Fig. 1*D*). Consistently, overexpression of SGCE significantly increased the mRNA and protein levels of FGF-BP1 in HCC1806 and HCC1937 cells (Fig. 1*E*).

### FGF-BP1 promotes TNBC stemness

**Figure 2:**
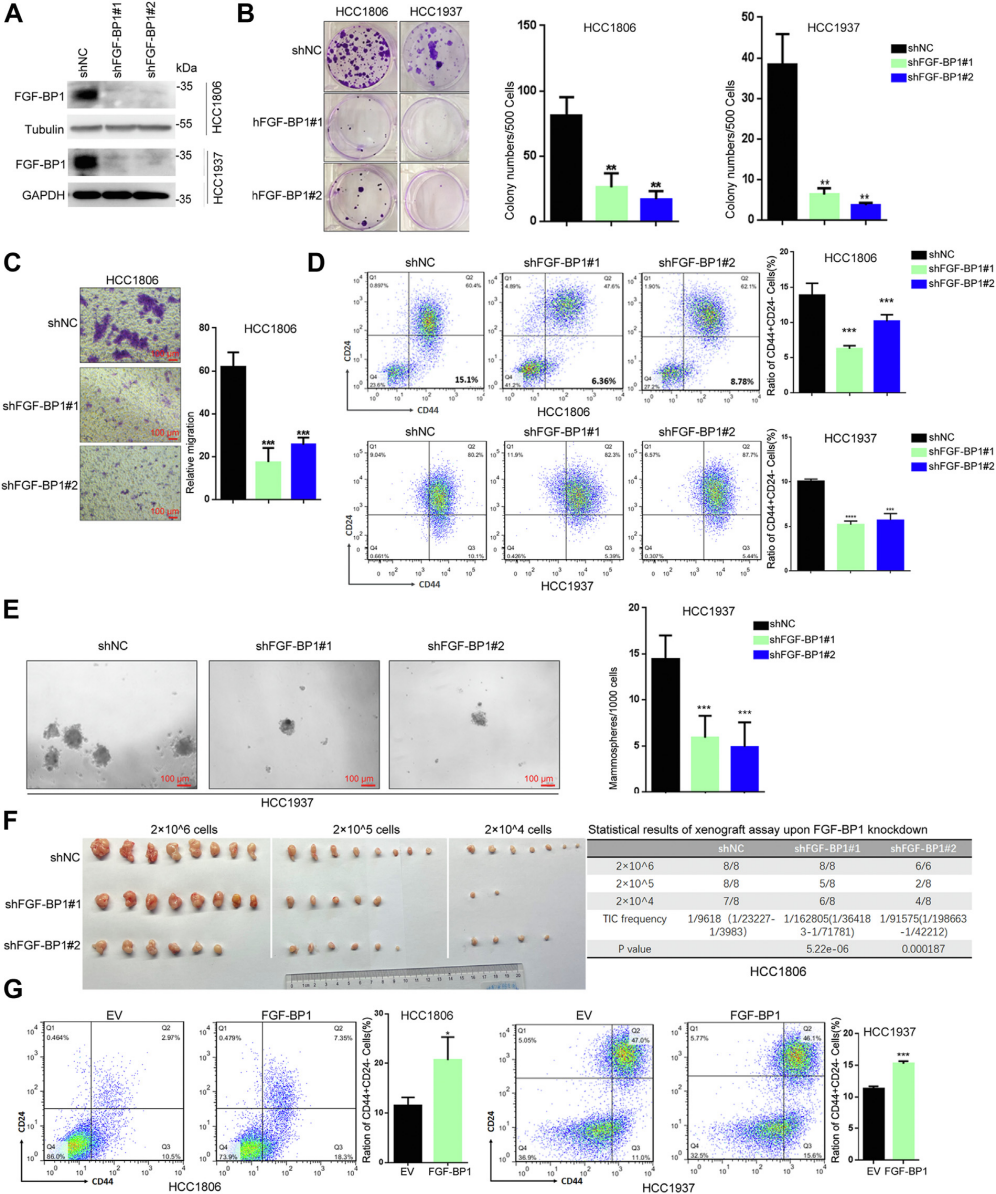
**FGF-BP1 promotes breast cancer cell stemness.***A*, FGF-BP1 knockdown efficiencies were detected by Western blot in HCC1806 and HCC1937 cells. *B*, clonal formation assay and their number calculations in FGF-BP1 depleted HCC1806 and HCC1937 cells. *C*, migration abilities of HCC1806 cells following FGF-BP1 knockdown and statistical results. *D*, assays of CD24^low^CD44^high^ population upon FGF-BP1 knockdown in HCC1806 and HCC1937 cells. *E*, tumorsphere assay upon FGF-BP1 knockdown in HCC1937 cells. *F*, xenograft assay (*left*) and statistical results (*right*) of xenograft assay upon FGF-BP1 knockdown using whole HCC1806 cell line. *G*, assays of CD24^low^CD44^high^ population upon FGF-BP1 overexpression in HCC1806 and HCC1937 cells were analyzed by FACS. Data are presented as the mean ± SEM. n = 3 per group except F. ∗∗*p* < 0.01, ∗∗∗*p* < 0.001 and ∗∗∗∗*p* < 0.0001 *versus* shNC (negative control) group or EV (empty virus control) group. FGF-BP1, fibroblast growth factor binding protein 1.

We previously reported that FGF-BP1 promotes breast cancer cell proliferation (21) and survival (28). Then, we wondered whether FGF-BP1 promotes breast cancer stemness. To reveal the physiological role of FGF-BP1 in breast cancer, we stably knocked down FGF-BP1 in HCC1806 and HCC1937 cells (Fig. 2*A*). Interestingly, knockdown of FGF-BP1 markedly reduced the clonal formation and migration abilities of HCC1806 and HCC1937 cells (Fig. 2, *B* and *C*). Besides, FGF-BP1 knockdown significantly decreased the CD24^low^CD44^high^ cell population (Fig. 2*D*) and reduced the number of spheres in HCC1937 (Fig. 2*E*). Furthermore, transplantations of HCC1806 cells with limiting dilution revealed lower frequencies of tumor formation upon FGF-BP1 knockdown, compared to control (Fig. 2*F*), which was consistent with SGCE knockdown (18). In agreement with this, FGF-BP1 overexpression dramatically increased the CD24^low^CD44^high^ cell population in both HCC1806 and HCC1937 cells (Fig. 2*G*). Altogether, our data identified that FGF-BP1 was closely related with tumor formation by promoting the stemness of TNBC cells.

### SGCE promotes TNBC stemness partially through FGF-BP1

**Figure 3:**
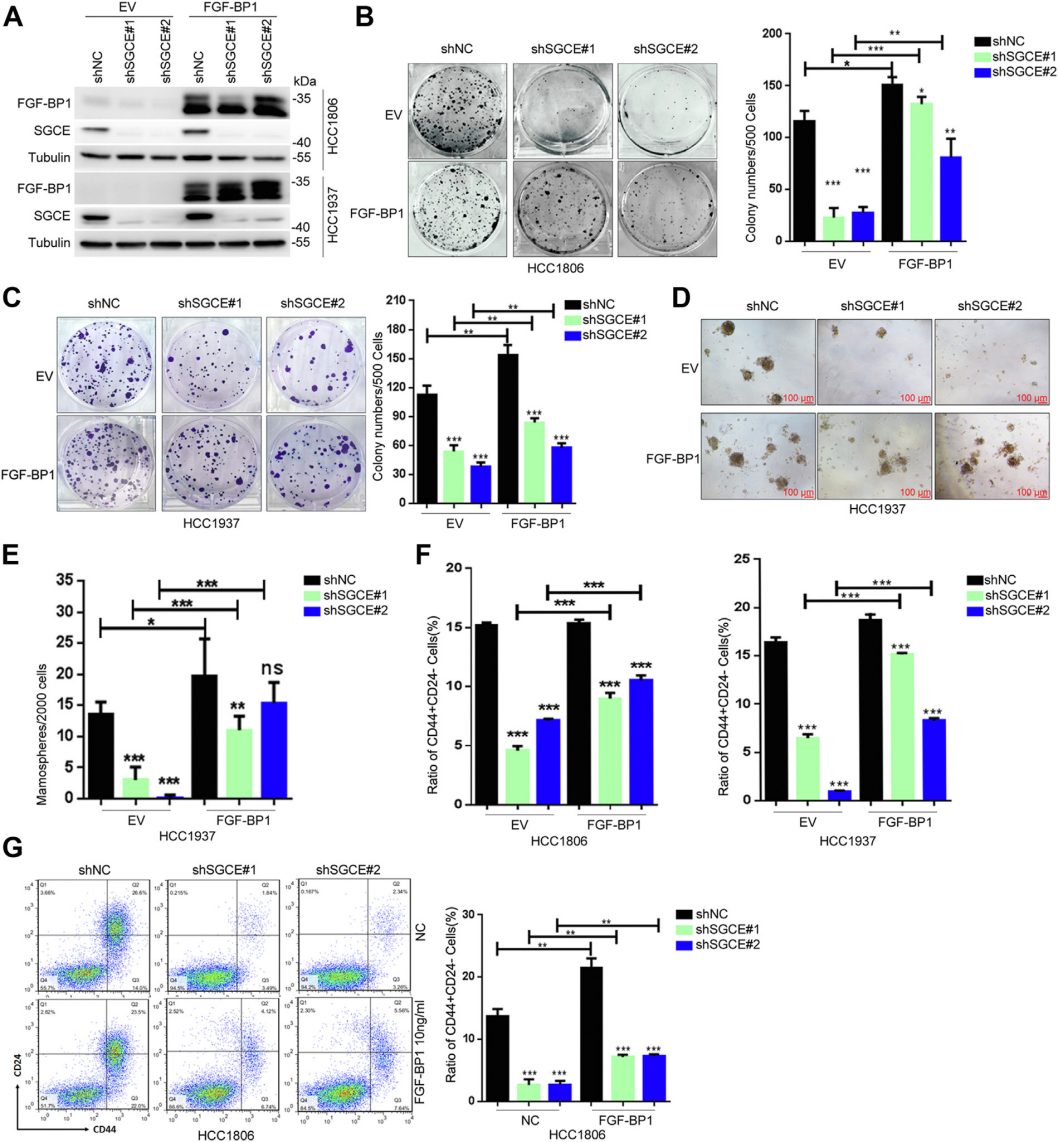
**SGCE promotes breast cancer cell stemness partially through upregulation of FGF-BP1.***A*, the efficiencies of FGF-BP1 overexpression in SGCE-deleted HCC1806 and HCC1937 cells were tested by Western blot. *B* and *C*, clonal formation assays showed that FGF-BP1 overexpression significantly rescued the colony formation inhibition induced by SGCE knockdown in HCC1806 and HCC1937 cells. *D* and *E*, tumorsphere assay upon FGF-BP1 overexpression in SGCE-deleted HCC1937 cells. *F*, assays of CD24^low^CD44^high^ population upon FGF-BP1 overexpression in SGCE-deleted HCC1806 and HCC1937 cells. *G*, SGCE-deleted HCC1806 cells were treated with 10 ng/ml FGF-BP1 for 48 h, then the HCC1806 cells were collected for CD24^low^CD44^high^ population assays. Data are presented as the mean ± SEM. n = 3 per group. ∗*p* < 0.05, ∗∗*p* < 0.01 and ∗∗∗*p* < 0.001 *versus* shNC (negative control) group or EV (empty virus control) group. FGF-BP1, fibroblast growth factor binding protein 1; SGCE, ε-sarcoglycan.

Given that SGCE promotes BCSCs partially by stabilizing EGFR (18), we explored the molecular mechanisms of SGCE involved in regulation of TNBC stemness. Clonal formation assay showed that the numbers of colonies was significantly reduced following SGCE knockdown, whereas overexpression of FGF-BP1 significantly restored the clonal formation abilities (Fig. 3, *A*–*C*). Furthermore, when FGF-BP1 was overexpressed in SGCE knockdown cells, the numbers of spheres were dramatically increased compared to SGCE knockdown cells (Fig. 3, *D* and *E*). Finally, flow cytometry analysis confirmed that enrichment of the CD24^low^CD44^high^ cell populations abrogated by SGCE interruption were significantly increased following FGF-BP1 overexpression (Fig. 3*F*). Thus, the above data indicate that FGF-BP1 was a downstream factor of SGCE-mediated stemness regulation.

As mentioned earlier, secreted FGF-BP1 is able be bound to the extracellular matrix to release fibroblast growth factor and is associated with tumor angiogenesis, cancer growth, and metastasis (19, 20). We wondered whether the recombinant FGF-BP1 protein could promote BCSC upon SGCE knockdown. As expected, the FGF-BP1 recombinant protein significantly reversed the decline of the CD24^low^CD44^high^ cell population induced by SGCE knockdown in HCC1806 cells (Fig. 3*G*). Together, targeting FGF-BP1 might improve the therapeutic effects of TNBC when SGCE protein levels are high.

### SGCE promotes TNBC stemness through FGF-BP1/FGF2/FGFR signaling

**Figure 4:**
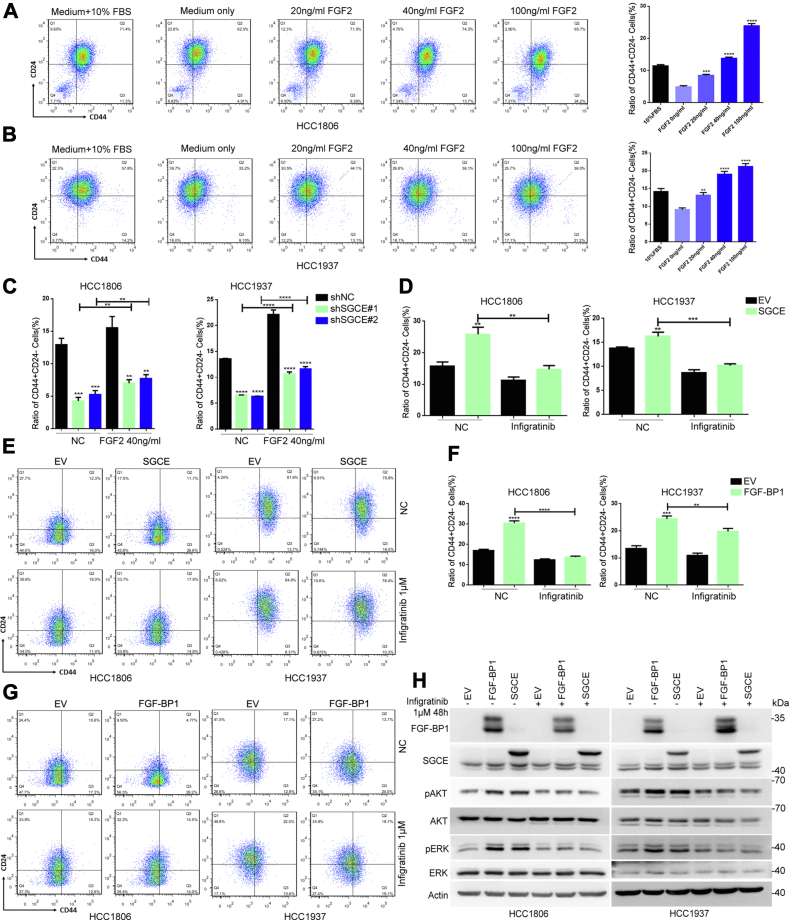
**SGCE and FGF-BP1 promote TNBC stemness through FGF-FGFR signaling.***A* and *B*, serum-starved HCC1806 and HCC1937 cells were treated with the indicated concentrations of FGF2 for 48 h, then the CD44^high^CD24^low^ stem cell populations were analyzed by FACS. *C*, after overnight serum starvation, the SGCE-deleted HCC1806 and HCC1937 cells were treated with 40 ng/ml FGF2 for 48 h, then assays of CD24^low^CD44^high^ population were analyzed by FACS. *D* and *E*, the SGCE-overexpression HCC1806 and HCC1937 cells were treated with 1 μm *Infigratinib* (FGFR inhibitor) for 48 h, FACS showed that *Infigratinib* decreased the CD44^high^CD24^low^ stem cell populations induced by SGCE overexpression. *F* and *G*, the FGF-BP1 overexpression HCC1806 and HCC1937 cells were treated with 1 μm *Infigratinib* for 48 h, then assays of CD24^low^CD44^high^ population were analyzed by FACS. *H*, the FGF-BP1 or SGCE overexpression HCC1806 and HCC1937 cells were treated with 1 μm *Infigratinib* for 48 h, then total AKT and phosphor-AKT (P-AKT), total ERK and phosphor-ERK (P-ERK) were examined by Western blot. Data are presented as the mean ± SEM. n = 3 per group. ∗∗*p* < 0.01, ∗∗∗*p* < 0.001 and ∗∗∗∗*p* < 0.0001 *versus* shNC (negative control) group or EV (empty virus control) group. FGF-BP1, fibroblast growth factor binding protein 1; SGCE, ε-sarcoglycan; TNBC, triple-negative breast cancer.

FGF-BP1 activates the FGF-FGFR signaling (19), which activates three dominant downstream pathways, RAS/MAPK, PI3K/AKT, and PLCγ, to maintain the self-renewal and pluripotency of stem cells (29). It has been reported that the expression levels of FGF2 are higher in TNBC patients with respect to non-TNBC patients (30). Therefore, we tested whether the recombinant protein FGF2 could promote TNBC stemness. As expected, FGF2 significantly improved the CD24^low^CD44^high^ cell populations in a dose-dependent manner (Fig. 4, *A* and *B*). Besides, flow cytometry analysis showed that FGF2 partially restored the reduction in the CD24^low^CD44^high^ cell population upon SGCE knockdown (Fig. 4*C*). In addition, treatment with the FGFR inhibitor *Infigratinib* decreased the CD24^low^CD44^high^ cell population induced by overexpression of SGCE or FGF-BP1 (Fig. 4, *D*–*G*), further suggesting the involvement of SGCE/FGF-BP1/FGF/FGFR signaling in TNBC stemness. Finally, we confirmed that overexpression of SGCE or FGF-BP1 upregulated p-ERK and p-AKT levels, whereas *Infigratinib* inhibited FGFR downstream signaling, including ERK and AKT signaling (Fig. 4*H*). Furthermore, the biological activity of FGF-BP1 and FGF2 were neutralized by *Infigratinib* (Fig. 4*H*), suggesting the potential for antibody-based therapeutic targeting. Thus, the above data suggested that SGCE regulated BCSCs through FGF-BP1/FGF2/FGFR signaling.

### SGCE upregulates the transcription of FGF-BP1 through Sp1

**Figure 5:**
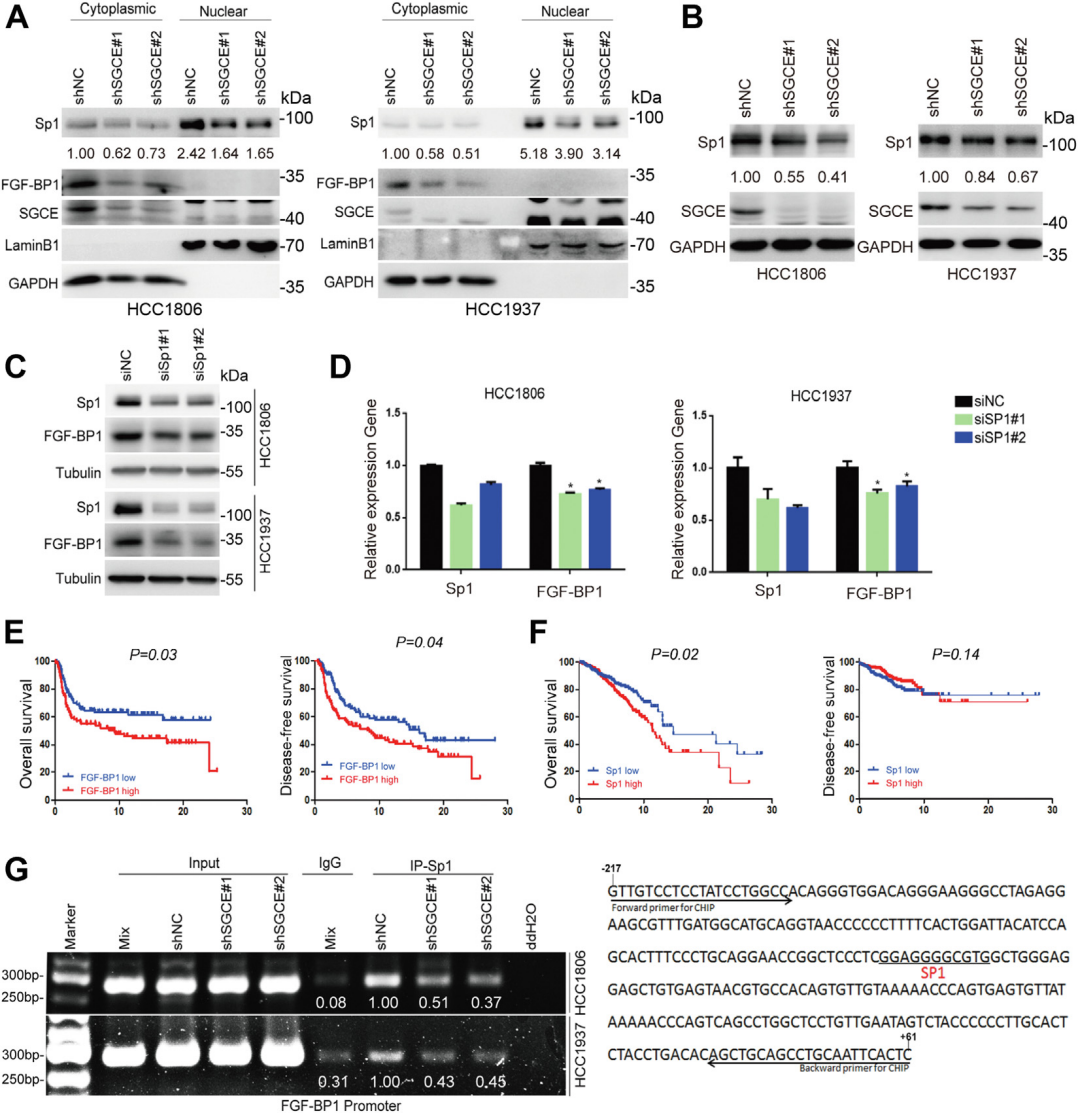
**SGCE promotes the transcription of FGF-BP1 through the transcription factor Sp1.***A* and *B*, SGCE knockdown decreased the total and nuclear Sp1 protein levels in HCC1806 and HCC1937 cells. *A* and *B*, the nuclear and cytoplasmic cell lysates (*A*), and whole-cell lysates (*B*) in the SGCE-deleted HCC1806 and HCC1937 cells were examined and quantified by Western blot. *C* and *D*, Sp1 was knocked down with different siRNAs in HCC1806 and HCC1937 cells. Western blot and RT-qPCR assay showed that Sp1 knockdown inhibited FGF-BP1 expression in HCC1806 and HCC1937 cells. *E* and *F*, results from METABRIC database showed the correlation of FGF-BP1 and Sp1 expression with overall survival and disease-free survival. *G*, SGCE knockdown significantly decreased endogenous Sp1 binding with FGF-BP1 gene promoter in HCC1806 and HCC1937 cells. ChIP-PCR assays were performed and qualified in the SGCE stable knockdown HCC1806 and HCC1937 cells. IgG was used as a negative control. The putative Sp1 binding site and PCR primers are shown on the right. Data are presented as the mean ± SEM. n = 3 per group. ∗*p* < 0.05 *versus* siNC (negative control) group. ChIP, chromatin immunoprecipitation; FGF-BP1, fibroblast growth factor binding protein 1; SGCE, ε-sarcoglycan.

**Supporting Figure S1:**
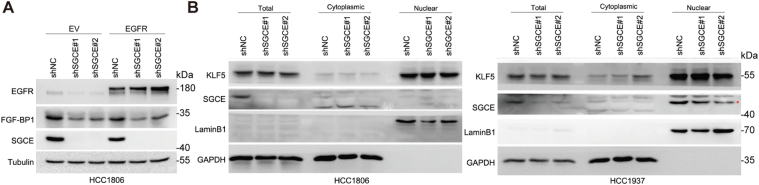
**RGFR and KLF5 might not regulate the transcription of FGF-BP1.***A*, Western blot analysis showed that overexpressed EGFR in SGCE knockdown could not rescue the decrease of FGF-BP1 in HCC1806 cells. *B*, cytoplasmic, nuclear, and whole-cell lysates were collected from HCC1806 and HCC1937 cells to detect KLF5 protein level by Western blot. *Red asterisk* means nonspecific band. FGF-BP1, fibroblast growth factor binding protein 1; KLF5, Krüppel-like Factor 5; SGCE, ε-sarcoglycan.

SGCE is known as a membrane protein stabilizing EGFR (18); therefore, we first tested whether SGCE regulated FGF-BP1 through EGFR, we found that EGFR overexpression could not rescue the decrease of FGF-BP1 induced by SGCE knockdown (Fig. S1*A*). Previous studies have shown that KLF5 promotes breast cell proliferation, survival, and tumorigenesis, partially by inducing *FGF-BP1* gene expression, activating ERK signaling, and stabilizing the MKP-1 phosphatase (21, 31, 32). Hence, we wondered whether SGCE regulates the expression of KLF5 and FGF-BP1. However, either nuclear localization or protein expression of KLF5 did not appear to be affected by SGCE knockdown (Fig. S1*B*). An Sp1 consensus site is essential for the basal activity of FGF-BP1 promoter (33, 34). Furthermore, both Sp1 and KLF5 are able to specifically bind to the GC boxes of FGF-BP1 promoter (35). We hypothesized that Sp1 may mediate the transcription of FGF-BP1 induced by SGCE overexpression. Here, we found that the total and nuclear Sp1 protein levels were reduced after SGCE knockdown (Fig. 5, *A* and *B*).

To further test whether endogenous Sp1 also regulates the *FGF-BP1* mRNA levels, we knocked down Sp1 by two well-characterized anti-Sp1 siRNA. We observed that the protein levels and mRNA expression of Sp1 and its downstream target gene *FGF-BP1* were markedly reduced in both cell lines (Fig. 5, *C* and *D*). Furthermore, an analysis including 995 patients with TNBC showed that FGF-BP1 and Sp1 high expression were associated with poorer overall survival (Fig. 5, *E* and *F*). We also found that FGF-BP1 high expression was also associated with shorter disease-free survival in patients with TNBC (Fig. 5*E*). The above data suggested that Sp1 and FGF-BP1 are potential prognostic markers. Finally, chromatin immunoprecipitation (ChIP) assays showed that Sp1 could specifically recognize the GC-box element in the proximal promoter region of FGF-BP1, whereas SGCE knockdown could inhibit the basal activity of FGF-BP1 promoter induced by Sp1 in HCC1806 and HCC1937 cells (Fig. 5*G*). Altogether, Sp1 is a key regulator of the FGF-BP1 transcription.

### SGCE increases Sp1 nuclear localization

**Figure 6:**
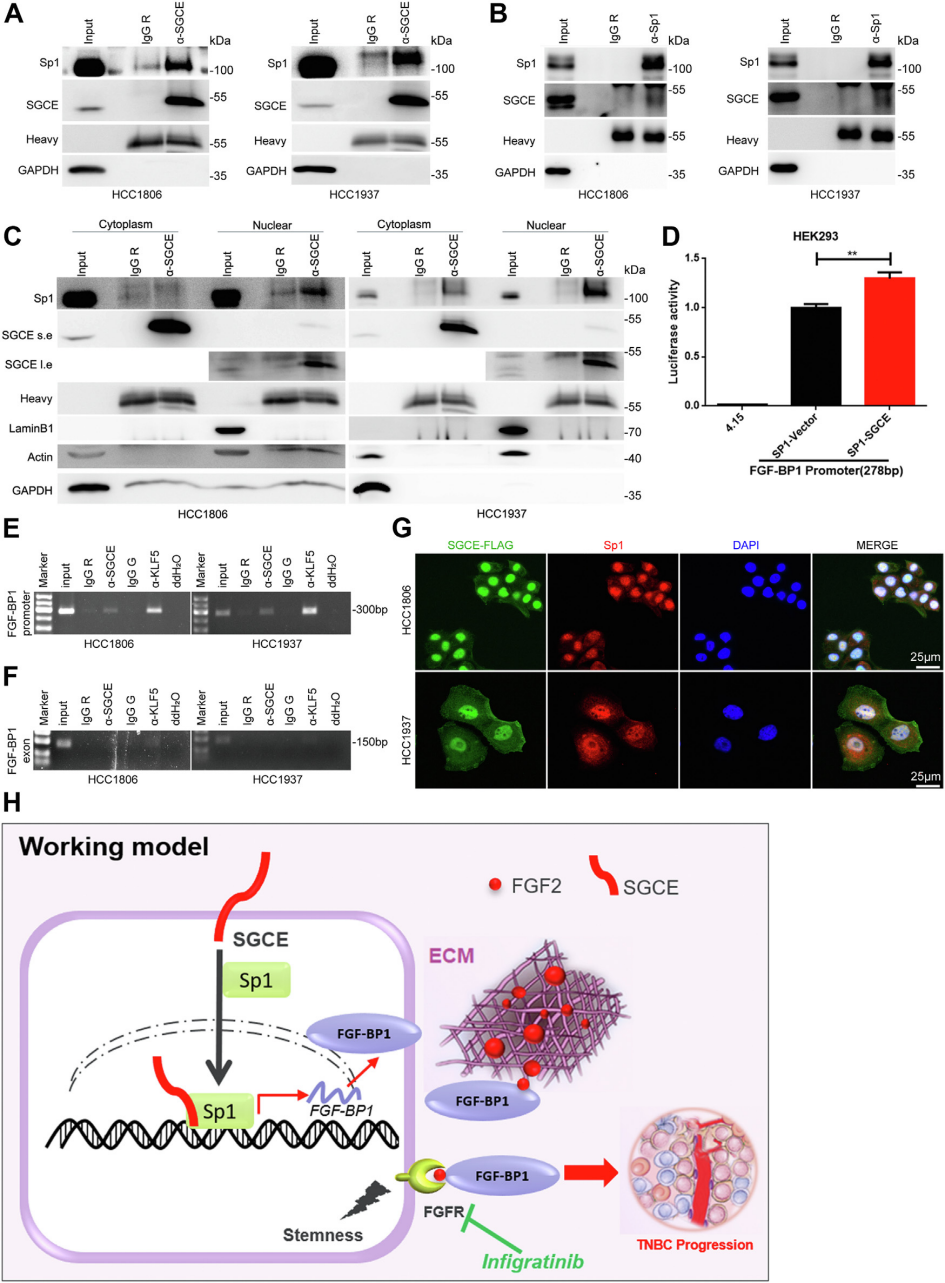
**SGCE and Sp1 form a transcription complex in the nucleus to activate FGF-BP1 transcription.***A* and *B*, coimmunoprecipitation of SGCE and Sp1 in HCC1806 and HCC1937 cells, and IgG R was used as a negative control. Heavy represents antibody heavy chain. *C*, SGCE mainly interacted with Sp1 in the nucleus of HCC1806 and HCC1937 cells. Cytoplasmic and nuclear cell lysates were collected from HCC1806 and HCC1937 cells to detect the interaction between SGCE and Sp1 by Western blot. *D*, transcriptional activity of FGF-BP1 was determined by luciferase reporter assay in HEK293T cells cotransfected with pGL4.15-luciferase plasmids and Sp1 or SGCE expression plasmid. pGL4.15-luciferase plasmid were used as negative control. *E* and *F*, ChIP-qPCR analysis showed that SGCE bound to the FGF-BP1 gene promoter. KLF5 was used as a positive control, and FGF-BP1 exon was used as a negative control. *G*, representative images of immunofluorescence analysis by confocal microscopy to detect colocalization of SGCE-FLAG and Sp1 in HCC1806 and HCC1937 cells. Scale bar, 25 μm. *H*, the hypothetical model according to this study. SGCE promotes TNBC stemness through the Sp1/FGF-BP1/FGF2/FGFR axis. Data are presented as the mean ± SEM. n = 3 per group. ∗∗*p* < 0.01. FGF, fibroblast growth factor; FGF-BP1, fibroblast growth factor binding protein 1; KLF5, Krüppel-like factor 5; SGCE, ε-sarcoglycan; Sp1, specific protein 1; TNBC, triple-negative breast cancer.

To explore the mechanism by which SGCE promoted the nuclear localization of Sp1, we first performed coimmunoprecipitation experiments and demonstrated that SGCE interacted with Sp1 in TNBC cells (Fig. 6, *A* and *B*). Next, we sought to determine how SGCE and Sp1 interact with each other and whether the interaction is required for their functions. Here, we found that SGCE interacts with Sp1 mainly in the nucleus as entire protein, but not in the cytoplasm (Fig. 6*C*). These findings suggested that the membrane protein SGCE could translocate into the nucleus as entire protein and form a transcription complex with Sp1. Indeed, luciferase reporter assays showed that the FGF-BP1 gene promoter was activated by the overexpression of SGCE and Sp1 in HEK293 cells (Fig. 6*D*). Consistently, ChIP assays indicated that SGCE could also specifically recognize the GC-box element in the proximal promoter region of FGF-BP1 (Fig. 6, *E* and *F*).

**Supporting Figure S2:**
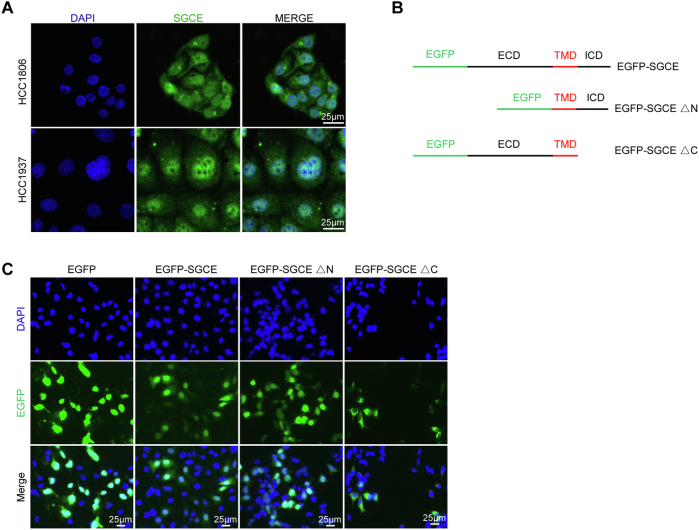
**C terminus played an important role in the nuclear translocation of SGCE.***A*, representative images of immunofluorescence analysis by confocal microscopy to detect localization of SGCE truncations in HCC1806 and HCC1937 cells. *B*, schematic diagram shows human SGCE and its truncation mutants. *C*, representative images of immunofluorescence analysis by confocal microscopy to detect localization of GFP-fused SGCE truncations in HEK293T cells. SGCE, ε-sarcoglycan.

Here, we found that SGCE protein was localized in the cytomembrane, cytoplasm, and nucleus of TNBC cells (Fig. S2*A*). Additionally, we fused SGCE with FLAG and performed immunofluorescence staining to detect the co-localization of SGCE-FLAG and Sp1 in HCC1806 and HCC1937 cells. The SGCE-FLAG protein colocalized with Sp1 in nucleus (Fig. 6*G*). Finally, we constructed two truncates of SGCE according to its domains and found that the C terminus of SGCE played an important role in nuclear translocation (Fig. S2, *B* and *C*).

Together, our data supported the notion that SGCE promotes breast cancer stemness partially by interacted with Sp1 to enhance the transcription of *FGF-BP1* (Fig. 6*H*).

## Discussion

Previously, we showed that SGCE is identified as a regulator of BCSCs self-renewal, chemosensitivity, and metastasis (18). Exploring these results, we focused our attention on the molecular mechanisms of SGCE involved in the TNBC stemness. Here, we report that SGCE and transcription factor PREP1 are required for the transcriptional regulation of FGF-BP1, thereby activating FGF2/FGFR signaling (Fig. 6*H*).

Sarcoglycan family of proteins are generally considered to assemble as dystrophin-associated glycoprotein complex on the plasma membrane. Here, we demonstrate that SGCE, a membrane protein, can translocate into the nucleus and interact with transcription factor Sp1. Similarly, the 4-pass transmembrane protein Tspan8 can achieve nuclear localization and promote breast stemness (36). Our previous study showed that SGCE functions as a sponge molecule for the interaction between EGFR and its E3 ubiquitination ligase (c-Cbl), and thus inhibits EGFR lysosomal degradation (18). Thus, FGF receptor may be regulated by SGCE. Further studies will be needed to illustrate the role of SGCE in regulating FGF receptor expression in TNBC cells.

This is the first report that SGCE can translocate into the nucleus, here we found that SGCE can interact with Sp1 in the nucleus and that both bind to the FGF-BP1 gene promoter. It was reported that the ectodomain of SGCE is cleaved under physiological conditions and that the C-terminal intracellular product is processed by the lysosome (37), similar to Notch (38). The extracellular region of SGCE is located at its N terminus and contains an immunoglobulin-like (Ig-like) domain (37), similar to iHog which responds to the active Hedgehog protein signal (39). SGCE may have the same characteristics as Notch and iHog. Here, we found that the C terminus of SGCE played an important role in nuclear translocation. However, what upstream signals regulate the nuclear translocation of SGCE, the potential function of SGCE in the nucleus, and the underlying molecular mechanisms require further investigation.

**Supporting Figure S3:**
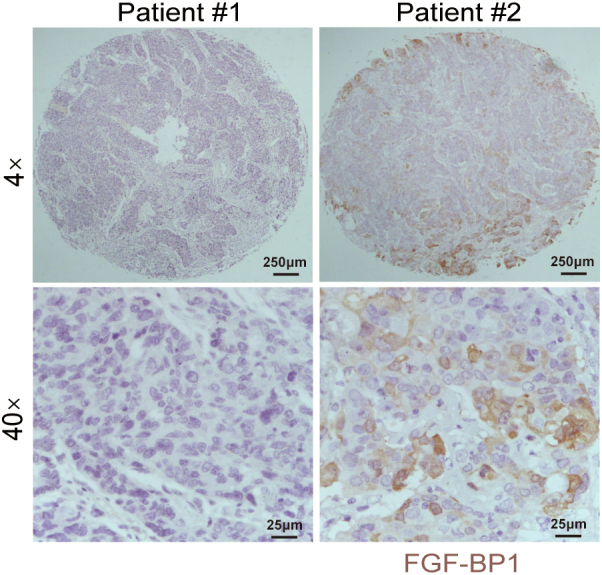
**FGF-BP1 protein expression levels in human TNBC.** Representative IHC results for FGF-BP1 in TNBC specimens. Scale bar, 25 μm. FGF-BP1, fibroblast growth factor binding protein 1; SGCE, ε-sarcoglycan; TNBC, triple-negative breast cancer.

Alterations in the FGF-BP1/FGF/FGFR signaling across the different subtypes of breast cancer have been described. FGF-BP1 is necessary for embryo survival, can regulate FGF-dependent vascular permeability in embryos, and is an angiogenesis switch in human cancer (40). In addition, FGF-BP1 can increase angiogenesis during skin wound healing and after hindlimb skeletal muscle ischemia injury (41). A number of studies have shown that FGF-BP1 has a tumor-promoting effect (19, 20, 40, 41, 42). Furthermore, Elena Tassi *et al.* (42) generated monoclonal antibodies against FGF-BP1 and found that the biological activity of FGF-BP1 is neutralized by antibody, suggesting the potential for antibody-based therapeutics. Whereas, SGCE and FGF-BP1 might have functional similarity and cross paths with each other. In the future, the underlying molecular mechanisms require further investigation. Given the important oncogenic function of secreted protein FGF-BP1 in breast cancer, FGF-BP1 has the potential to be a serum biomarker and therapeutic target. We tested the protein level of FGF-BP1 in human TNBC clinical samples by immunohistochemical staining. Remarkably, FGF-BP1 positivity was observed in 43.3% (13 of 30) of TNBC samples (Fig. S3).

FGFR aberrations are common in a wide variety of cancers (18% FGFR-aberrant in breast cancer), with the majority being gene amplifications or activating mutations (43). Thus, FGFR inhibition has been recently considered as a promising therapeutic option for breast cancer patients. Therapies targeting FGFRs have shown promising results in many cancer types and been approved for the treatment of urothelial carcinoma and cholangiocarcinoma (44, 45). Adaptive or intrinsic resistance is a common problem limiting the therapeutic efficacy of FGFR inhibitors. For this reason, we need to identify breast cancer patients with FGFR alterations who might better respond to treatment. Here, we found that SGCE, Sp1, FGF-BP1, and FGFRs are potential therapeutic targets for TNBC. TNBC patient with SGCE and FGF-BP1 positive might be benefit from targeting FGFRs treatment. Unfortunately, we failed to obtain an antibody for IHC to analyze the expression of SGCE in clinical breast cancer samples. In the future, SGCE conditional knockout mice will be required to confirm our results.

In summary, we demonstrated that SGCE can translocate into the nucleus and interact with transcription factor Sp1, positively regulating the transcription of FGF-BP1. The SGCE/Sp1/FGF-BP1/FGF2/FGFR axis promotes stemness in TNBC and serves as a potential therapeutic target.

## Experimental procedures

### Materials

All primers, antibodies, and agents (kits) can be found in Tables S1–S3.

### Cell culture and transfection

HCC1806, HCC1937, and HEK293T cells were purchased from American Type Culture Collection and validated *via* short tandem repeat analysis. HCC1806 and HCC1937 were cultured in RPMI-1640 medium (Gibco) containing 10% fetal bovine serum (ExCell Bio, FSD500). The HEK293T cell line was cultured in Dulbecco's modified Eagle's medium (Gibco) supplemented with 10% fetal bovine serum. All cells were maintained in an incubator with 5% CO_2_ at 37 °C.

### Coimmunoprecipitation and Western blot

Cells were washed with PBS and lysed in ice-cold lysis buffer (lysis buffer: 150 mM NaCl, 1 mM ETDA pH 8.0, 50 mM Tris-HCl pH 7.4, 1% Triton X-100) for 30 min with protease inhibitor (MCE, HY-K0010). Cell lysates were incubated with the indicated antibodies for 36 h at 4 °C, followed by incubation with protein A/G magnetic beads (MCE, HY-K0202) for 6 h at 4 °C. The beads were washed with cell lysis buffer three to five times. Finally, the beads were boiled in 2× SDS loading buffer at 95 °C for 15 min. The eluents were analyzed by Western blot. Lysis samples were separated by sodium dodecylsulphate polyacrylamide gel electrophoresis (SDS-PAGE), transferred onto polyvinylidene fluoride membranes, blocked with 5% nonfat dried milk for 1 h, incubated with the indicated primary antibody at 4 °C overnight and with secondary antibody conjugated HRP for 1 h, and then detected with a chemiluminescent HRP substrate (US EVERBRIGHT, s6009-500 ml, China).

### Mammosphere assay

The HCC1806 and HCC1937 cells were plated in ultralow attachment 96-well plates with EpiCult-B Basal Medium (Human) and Epicult-B Proliferation Supplement (Human) with hydrocortisone and heparin. The mammosphere was calculated after 10 to 14 days.

### Flow cytometry

Cells were grown in 6-well plates (10^5^ cells per well). After 48 h, the cells were digested, counted, and stained with the following antibodies: anti-CD44-FITC (BD, 1:100), anti-CD24-PE (BD, 1:100), and anti-7AAD (1:500). The details have been described (31).

### Quantitative RT-PCR

Total RNA from cells and tumor tissues was extracted using Trizol (15596–026, Invitrogen) based on the manufacturer’s instructions. RNA was resuspended in RNase free water. Complementary DNA (cDNA) was produced from 1 μg of RNA using the ExScript RT Reagent Kit (RR037A, Takara) per the relevant instructions. The cDNA samples were subjected to RT-qPCR using SYBR mix (Invitrogen, 4472908).

### Clonal formation assay

The HCC1806 and HCC1937 cells were seeded into 6-well plates at a density of 500 cells per well. Cells were cultured for 10 days, with the medium changed every 3 days. The cells were then fixed in 4% paraformaldehyde (PFA) and stained with 0.2% crystal violet.

### Xenograft assay

Nude female mice (aged 8 weeks) were used to study tumorigenesis ability (18). The HCC1806 cells suspended in a 1:1 mixture of PBS, and Matrigel (total volume of 100 μl) were injected into the mammary fat pads. After 4 weeks of adaptation, the presence of palpable tumors was examined. The number of tumor-initiating cells was calculated using the extreme limiting dilution analysis web interface (http://bioinf.wehi.edu.au/software/elda/). The animal experiment was approved (SMKX-20160305-08) by the Animal Ethics Committee of the Kunming Institute of Zoology, CAS.

### Plasmids, siRNAs, and transfection

All transfections for plasmids and siRNAs were performed using Lipofectamine 2000 (Invitrogen) according to the manufacturer’s instructions. In brief, cells were grown to 50 to 60% confluence and transfected with the respective plasmids or siRNA. Plasmids or diluted siRNA and Lipofectamine 2000 were added to separation tubes containing serum-free medium and incubated at room temperature for 5 min. The contents of the two tubes were then mixed, incubated at room temperature for 20 min, and distributed onto the respective cell culture dishes. The cells were incubated in 5% CO2 at 37 °C for 48 h for further experiments. All chemically synthesized siRNAs were purchased from RiboBio and transfected at a final concentration of 20 nM.

### shRNA transduction

Cell lines that stably expressed the specific shRNA or nontargeted control shRNA (sh-control) were constructed using the PLKO.1-based lentiviral shRNA technique. The shRNA plasmid, along with PMD2.G and psPAX2 (4:1:3), was transfected into the 293T cells to produce lentiviral particles. The HCC1806 and MDA-MB-231 cells were infected with lentiviruses expressing the shRNA constructs with polybrene after cells were seeded into 6-well plates for 16 to 24 h. Fresh medium was added after 24 h, with the cells then selected with puromycin to obtain cell lines stably expressing shRNA.

### Immunofluorescence

The HCC1806 and HCC1937 cells were seeded on coverslips in 12-well plates (50,000 cells per well) and fixed in 4% PFA at 4 °C. Cells were then blocked with 0.1% Triton X-100 in 5% bovine serum albumin for 1 h, followed by incubation with primary antibodies against Sp1 (Santa Cruz, SC-59 1:100) and SGCE (Sigma, HPA074790, 1:100) overnight at 4 °C. and Alexa Fluor 488- or 594-labeled secondary antibodies (Abclonal, 1:1000) were used to treat cells for 1 h. Finally, coverslips were mounted with 4′,6-diamidino-2-phenylindole (Vector Laboratories) and observed *via* laser-scanning confocal microscopy (ZEISS).

### Migration assay

After cells grew exponentially, they were seeded into 24-well culture inserts with 8 μm pores. After 24 h, a cotton swab was applied to clean the cells on the upper surface of the filters. Cells on the lower filter surface were defined as the invaded cells. For easier visualization, the cells were fixed in 4% PFA and stained with 0.2% crystal violet. In the invasion assay, before seeding the cells, the inserts were first coated with Matrigel (BD) for 24 h, after which the cells and Matrigel were removed with a cotton swab.

### Chromatin immunoprecipitation

ChIP assays were performed using the HCC1806 and HCC1937 cells following a protocol as described (46). The diluted DNA–protein complex (25 μg protein) was incubated with anti-Sp1 antibody (Santa Cruz, SC-59, 6 μg), SGCE (Sigma, HPA074790, 6 μg) overnight at 4 °C in the presence of herring sperm DNA and protein A/G beads or anti-Flag magnetic beads. The primers for the FGF-BP1 gene promoter region were 5′- GTTGTCCTCCTATCCTGGCCA-3′ (forward) and 5′- GAGTGAATTGCAGGCTGCAGCT -3′ (reverse), and the product was 278 bp.

### Dual luciferase assays

The FGF-BP1 promoter was amplified using normal human HCC1806 cDNA as a template. The PCR products were cloned into the pGL4.15-Basic vector. The constructs were confirmed by DNA sequencing. HEK293 cells were seeded in 48-well plates at 3 × 10^4^ cells per well. After 16 to 24 h of culture, the cells were transfected with the FGF-BP1 promoter reporter plasmid (0.15 μg/well) and the Renilla internal control plasmid (0.01 μg/well), together with Ptomo-SGCE plasmid (0.15 μg/well) or Plvx-3× Flag Sp1 plasmid (0.15 μg/well) or negative control plasmid in triplicate. At 48 h after transfection, luciferase activities were measured using the dual luciferase reporter assay system (Promega).

### Immunohistochemical staining

Human breast cancer specimens were obtained from 30 patients who had undergone surgical resection between 2006 and 2015 at the First Affiliated Hospital of Kunming Medical University. Surgical specimens were formalin fixed, sectioned, stained with hematoxylin and eosin, and examined by microscopy. Diagnoses were made by experienced pathologists and graded using standard histological and modified Scarff–Bloom–Richardson criteria. Human breast cancer specimen slides were incubated at 60 °C for 2 h and subjected to antigen retrieval prior to conventional IHC. Anti-FGF-BP1 primary antibody was used at a 1/1000 dilution. Staining patterns were interpreted by two pathologists with no prior knowledge of the clinicopathological parameters. Immunostained slides were evaluated under a light microscope.

### Statistical analysis

Student’s *t* test (2-tailed) was used to compare differences between two groups. One-way ANOVA was used to analyze the differences among multiple groups. Data are presented as the means ± standard deviation (SD). *p* values of < 0.05 were considered significant. All data were analyzed using GraphPad Prism 6 (GraphPad Software Inc). Statistical data were calculated using GraphPad Prism 6 (GraphPad Software Inc)

## Data availability

Other data supporting the study findings are available from the corresponding author upon reasonable request.

## Supporting information

This article contains supporting information.

## Conflict of interest

The authors declare no conflicts of interest with the contents of this article.
